# In Vivo Glutathione S-Transferases Superfamily Proteome Analysis: An Insight into *Aedes albopictus* Mosquitoes upon Acute Xenobiotic Challenges

**DOI:** 10.3390/insects13111028

**Published:** 2022-11-07

**Authors:** Siti Nasuha Hamzah, Silas Wintuma Avicor, Zazali Alias, Sarah Abdul Razak, Siti Khadijah Mohd Bakhori, Ting Chuan Hsieh, Nurin Nazifa Syanizam, Salinah Abdul Farouk

**Affiliations:** 1 School of Biological Sciences, Universiti Sains Malaysia, George Town 11800, Penang, Malaysia; 2 Entomology Division, Cocoa Research Institute of Ghana, New Tafo-Akim P.O. Box 8, Ghana; 3 Institute of Biological Sciences, Faculty of Science, Universiti Malaya, Kuala Lumpur 50603, Malaysia; 4 School of Physics, Universiti Sains Malaysia, George Town 11800, Penang, Malaysia

**Keywords:** glutathione S-transferase, *Aedes albopictus*, metabolism detoxification, peptides, xenobiotic challenge

## Abstract

**Simple Summary:**

Glutathione S-transferases (GSTs) are a superfamily of enzymes that play a crucial part in phase II detoxification reactions. In many cases, GSTs have been implicated in toxicological challenges by association only (i.e., detected using model substrates or due to an increase in GST activity) and not through the identification of the individual peptides involved. In this study, the aim was to identify the GSTs induced by acute insecticide challenges up to the isoform or peptide level and the detoxification mechanisms involved in *Aedes albopictus* larvae. Upon permethrin and malathion inductions, the GST-based peptides were identified, and the functional characteristics as well as protein-protein interactions were analyzed along with the quantification of the abundance. Twenty-four GST peptide isoforms were identified under seven different classes with a highly significant protein-protein interaction between GST groups and within other detoxification metabolism-related peptides (*p*-value < 1.0 × 10^−16^). Overall, our findings indicate that out of seven GST classes, only Delta and Epsilon GSTs were mainly involved in the detoxification mechanism via the direct glutathione metabolism or the sequestration of the insecticides, as well as providing the protection of sulfhydryl groups in enzymes against oxidative stress induced by insecticide exposure.

**Abstract:**

In this study, the induction of glutathione S-transferase (GST) enzymatic activities in *Aedes albopictus* under 24 h of xenobiotic challenges was investigated. From LCMS analysis, 23 GST isoforms were identified under Delta, Epsilon, Sigma, Zeta, Omega, and Iota classes, together with one GSTX1-1 isoform, in both treated and untreated samples. Using STRING 11.5, the functional enrichment network of Gene Ontology (GO) analysis, the identified peptides were found to be involved in the glutathione metabolic biological process (GO:0006749, *p*-value: 1.93 × 10^−29^), and the molecular functions involved are due to glutathione transferase (GO:0016848, *p*-value: 2.92 × 10^−8^) aside from carbon-halide lyase activity (GO:004364, *p*-value: 1.21 × 10^−31^). The Protein-Protein Interaction (PPI) network (STRING 11.5) showed significant interactions within the GST superfamily and some of the GST classes interacted with other proteins among the input domain of the identified peptides (*p*-value < 1.0 × 10^−16^). In TMT labeling for the quantification of peptide abundance, isoforms from Delta (GSTD1-2, GSTD1-3, GSTD1-4) and Epsilon (GSTE3-1, GSTE4-2) were found to be overexpressed (between 1.5-fold and 2-fold changes). In the PPI analysis, 12 common enriched pathways of Kyoto Encyclopedia of Genes and Genomes (KEGG) were found to be intercorrelated with the identified GSTs at PPI enrichment *p*-value < 1.0 × 10^−16^. Overall, this study indicates that distinct GST enzymes, which were identified up to their specific protein isoforms, are involved in the metabolic mechanisms underlying xenobiotic stress.

## 1. Introduction

Glutathione S-transferases (GSTs: E.C.2.5.1.18) represent a major group of detoxification enzymes, comprised of numerous hydrophobic toxic compounds, and are multifunctional dimeric proteins crucial in drug metabolism, xenobiotic and endobiotic biotransformation, intracellular transport, the biosynthesis of hormones, and the metabolism of xenobiotics [1,2,3,4,5]. Among these, GSTs play a predominant role in providing protection against electrophiles and oxidative stress products [5].

The mammalian GSTs have been categorized into eight classes which are Alpha, Mu, Pi, Theta, Sigma, Zeta, Kappa, and the microsomal class [5,6]. Specific interest has been shown towards GSTs due to their role in metabolic enzyme-based insecticide resistance as various insects have demonstrated elevated GST enzymatic activity [6,7]. Studies have emphasized the action of insect GSTs in insecticide resistance, particularly dichlorodiphenyltrichloroethane (DDT) and pyrethroid resistance [2,4,8,9,10]. The role of GSTs in the dehydrochlorination of DDT is the primary route of its detoxification and serves as the most common DDT resistance mechanism in mosquitoes [11,12].

A total of six cytosolic GST classes have been identified in insects including mosquitoes which are Delta, Sigma, Epsilon, Omega, Theta, and Zeta [13,14,15,16]. Among these classes, the Delta and Epsilon classes are apparently the primary classes involved in the metabolic detoxification of xenobiotics and have been identified to be responsible for the propagation of insecticide resistance [6,10,13,15]. Penilla et al. [17] reported that there is an increase in GST activities under continuous pyrethroid selection in field strains of *Anopheles albimanus*. The metabolic resistance to DDT in *Anopheles* spp. larvae and adults is primarily conferred by an increase in GST activity [15,18].

The species of interest in this study is *Aedes albopictus*, which acts as the secondary dengue vector in Malaysia as well as being the pathogen carrier of various other viruses [19]. Dengue poses a serious health threat in Malaysia, and the study of the species is, therefore, pertinent to reducing the burden of the disease. *Aedes* mosquito management is the sole strategy that can be used since a stable dengue vaccine is yet to be established [20,21]. Utilizing pesticides, eliminating larval breeding grounds, and using spatial repellents are the primary methods for controlling *Ae. albopictus* [22,23,24]. Unfortunately, mosquitoes metabolize chemicals using their defense mechanisms as a result of the continuous, intensive, and broad application of insecticides, which eventually results in resistance to the insecticides, a rapidly emerging phenomenon [22,25]. The selection pressure of insecticides and the inheritability of resistance among generations of vector populations are some other aspects that undermine the efforts to control vectors [10,26].

Insects evolve xenobiotic resistance primarily through improving the metabolic efficiency of their detoxification systems and/or decreasing the sensitivity of the xenobiotic target site [26]. Metabolic-based resistance occurs when the amount of insecticide that reaches the target site is reduced due to an enhanced rate of xenobiotic metabolism caused by the actions of enzymes [27]. Resistance involving the enzymatic activities of metabolic enzymes is of concern due to the fact that the enzymatic activities are not constant and differ according to different strains from different locations which are caused by various factors. For integrated vector control programs to be successful yet sustainable, it is essential to understand the underlying mechanisms of pesticide resistance [24]. A biochemical assay provides information regarding the total amount of protein and the catalytic behaviors of the target enzyme. Proteomic analysis on the other hand enables the identification and quantification of the different expression levels of the enzymes. Considering insecticides from the pyrethroid and organophosphate groups are frequently employed in programs to control mosquitoes, the intended outcomes of this study were to investigate the effect of 24 h of acute insecticide treatments on *Ae. albopictus* in terms of GST enzymatic activities in order to identify the toxicological challenge mechanisms in *Ae. albopictus* larvae. Subsequently, the different classes and isoforms of GST enzyme proteomes, as well as their expression levels based on their peptide abundance upon the acute 24 h treatment with malathion and permethrin, were observed.

## 2. Materials and Methods

### 2.1. Mosquito Strains

An established 284th generation of a susceptible strain of *Ae. albopictus* was obtained from the Vector Control Research Unit (VCRU), Universiti Sains Malaysia. The 4th instar larvae of the susceptible *Ae. albopictus* were used as the acute insecticide-treated strains and the untreated control strains.

### 2.2. Determination of Toxicity Parameters and Insecticide Treatment

The susceptible VCRU strain was subjected to the standard World Health Organization (WHO) larval bioassay procedure [28,29] to determine the sub-lethal concentrations (LC50) of malathion (96.0% purity) and permethrin (96.9% purity). Batches of 25 early 4th instar larvae of the laboratory susceptible strain were transferred to a 500 mL plastic cup containing a mixture of seasoned water and insecticide solution with a final volume of 250 mL. Ethanol was used as the solvent to dilute the insecticides. Seasoned water containing 1% ethanol and larvae summing up to 250 mL was used as control. A range of concentrations in four replicates against the yielded mortality ranging from 0% to 100% within 24 h was plotted using Probit analysis in SPSS software version 24 [30] to obtain the LC50 value [29]. For the acute treatment, the susceptible larvae were exposed to the determined malathion and permethrin LC50 for a period of 24 h. Every treated larva that survived was collected and used in further analysis.

### 2.3. Protein Determination and Enzyme Assays

The early 4th instar larvae of the susceptible strains and larvae which survived acute treatment with the insecticides for 24 h were individually homogenized in 200 μL of seasoned water on ice and centrifuged at 14,000 rpm, 4 °C for 30 s, and the supernatant was used as an enzyme source in the assays. At least 100 individual replicates were used in the assays. The assays were performed in a 96-well microplate on ice and the absorbance (optical density [OD] values) was measured on the microtiter plate reader (Thermo Fischer Scientific) with Magellan data analysis software. The total protein content (mg) was determined using the Bradford protein assay with bovine serum albumin (BSA) as a standard to normalize the activities for protein concentration [31]. The GST activities toward the model substrates were conducted according to Hemingway [32] and Hamzah and Alias [33]. The GST conjugation activities with 1-Chloro-2,4-dinitrobenzene were determined at 340 nm. The specific activity was calculated in μmol/min/mg protein by following Beer’s Law, A= εcl where A is the absorbance; ε is the extinction coefficient; c = concentration; and l is the path length. Then the total enzyme activity was expressed as μmol/min at 25 °C [34].

### 2.4. Trypsin Digestion and Tandem Mass Tag (TMT) Labelling

A tandem mass tag (TMT) was used as a chemical label for mass spectrometry (MS)-based identification and to quantify the identified peptides. Briefly, the supernatant of homogenized susceptible, acute permethrin-treated and malathion-treated larvae were lyophilized and re-suspended in a Radioimmunoprecipitation assay (RIPA) buffer. The samples were concentrated by precipitation using the Total Protein Precipitation Kit (ITSIBIO, USA). Each sample was assayed for protein content using the total protein assay kit (ITSI Biosciences). The dried protein pellet was re-suspended in a lysis buffer. An equal amount of protein (20–100 µg) from each sample was reduced with tris(2-carboxyethyl) phosphine (TCEP) and alkylated with iodoacetamide. The precipitated samples were then re-suspended in 100 mM triethylammonium bicarbonate (TEAB), and trypsin digestion was performed overnight. The digested samples were individually labeled with 3 different TMT reagents (6 plex amine reactive tags kit) according to the manufacturer’s protocol (Thermo Scientific). The fractions were desalted by using C18 zip tips and dried down.

### 2.5. Liquid Chromatography Mass Spectrometry

Liquid Chromatography-Mass Spectrometry (LCMS) was performed according to the manufacturer’s manual (Thermo Fisher) by using an Orbitrap Fusion mass spectrometer (Thermo Fisher). It was equipped with a 100 µ × 20 mm C18 100 Å 5U reverse-phase trap for online desalting and a 75 µ × 150 mm C18 200 Å 3U reverse-phase column for peptide separation. Subsequently, 1 μL of the peptide mix was loaded onto the reverse-phase column at an isocratic flow rate of 300 mL/min and a gradient of 0.1% formic acid (A) and 100% acetonitrile (B). The eluents gradient used was 5% to 35% B for 60 min. The high-mass accuracy MS full spectra data were obtained in the data-dependent mode with the Orbitrap MS (OTMS). The resolution was set to 120,000 at MS1 with a scan range of 310–1800 m/z, an automatic gain control (AGC) target of 400,000, and a maximum injection time of 50 milliseconds. The method consisted of a 3 s top-speed mode where precursors were selected for a maximum 3 s cycle. Only precursors with an assigned monoisotopic m/z and a charge state of 2–7 were further analyzed for MS2. All precursors were filtered using a 20 s dynamic exclusion window and an intensity threshold of 5000. The MS2 spectra were analyzed with an ion trap MS (ITMS) set with a rapid scan rate at a resolving power of 60,000, an AGC target of 100, a 1.6 m/z isolation window, and a maximum injection time of 250 milliseconds. The precursors were fragmented by collision-induced dissociation (CID) and high-energy collision dissociation (HCD) at a normalized collision energy of 30% and 28%, respectively. The top 10 fragmented ions from MS2 were fragmented again to produce the MS3 spectrum to increase the TMT reporter ion population. Every MS3 precursor population was fragmented by HCD at 65% collision energy.

### 2.6. Analytical Statistics

The data obtained in WHO bioassays were statistically analyzed by using the Probit analysis computer program SPSS version 24 [30] to determine LC50 values [29]. Adjustments were made using Abbott’s formula [34] if the control mortality ranged from 5% to 20%. If the pupation exceeded 10%, the test was rejected [29]. The LC50s of permethrin and malathion obtained for the control strain were used further as the dose for the acute-insecticide-treatment effect study. The mortality percentages and enzymatic activities of GST were tested for normality as well as variance homogeneity by using Komolgorov–Smirnov and Levene’s tests, respectively. The GST enzymatic activity data were presented as mean ± standard deviation (S.D.). One sample *t*-test (*T*-test) was applied to compare the total protein content and particular enzyme expression levels between each acute insecticide-treated strain with the susceptible strain only.

### 2.7. Querying Domain and Interaction Information

For proteomic analysis, the GST raw data files were searched against the most recent database for *Aedes* downloaded from Uniprot by using the MudPIT option in Proteome Discoverer 2.1 (Thermo Scientific) and the Sequest HT search algorithm. Only peptides identified with high confidence were considered and used for protein identification. Trypsin was the selected enzyme allowing for up to two missed cleavages per peptide. Carbamidomethylation of cysteine, N-terminal TMT3-plex, and Lysine TMT3-plex were used as a static modification whereas the oxidation of Methionine and the TMT3-plex of Threonine were used as variable modifications. The group peptide abundances were generated using Proteome Discoverer 2.1 software coupled with the Sequest HT search algorithm. After the Peptide and Protein Quantifier node classified the peptide groups, it calculated protein abundances for the samples as the simple summation of its associated peptide group abundances. Precursor ion area detection presented the average peak areas of the top N unique peptides for each protein, usually set to 3 by default. The node creates the Abundance Counts column on the Proteins page, which shows the number of peptide group abundances used for calculating abundances. Finally, the node calculated the ratios (fold-change) by dividing the abundance values of the associated sample groups (susceptible, malathion-treated, and permethrin-treated samples). Thus, a TMT ratio ≥ 1.2 was classified as upregulated, and ≤0.80 was classified as downregulated. A fold-change from 0.81 to 1.19 was considered a moderate-to-no change. Ratios above 100 exceeded the maximum allowed threshold that was reasonably expected.

### 2.8. Gene Ontology (GO) and Kyoto Encyclopedia of Genes and Genomes (KEGG) and Protein-Protein Interaction (PPI) Analysis

Uniprot (https://www.uniprot.org/ accessed on 24 May 2022) was used to obtain information on the identified protein domains. Gene Ontology (GO), the Kyoto Encyclopedia of Genes and Genomes (KEGG), and the Protein-Protein Interaction (PPI) network were integrated using STRING analyses. The interaction between the identified proteins was determined by using STRING 11.5 at an interaction coefficient of 0.4 as the minimum standard. Protein interactions with confidence coefficients larger than 0.4 (medium confidence) were regarded as relevant because it was the most often employed analytic criterion in STRING. Twenty-four identified protein accessions generated from the MudPIT option in Proteome Discoverer 2.1 (Section 2.7) were used in the STRING analysis. The accessions are J9HXZ8, J9HHL7, Q17MA9, A0A1S4EXN6, Q6PTY1, A0A0N8ES64, Q17MB8, Q16SH6, A0A023ENG1, Q16SH7, Q170C6, Q5PY78, Q170C9, A0A0P6IV26, Q170C7, Q16P53, J9E9C0, A0A1S4G560, A0A0P6J0T5, Q1HQK1, Q16P79, Q16P80, Q0C791, and Q16NL9. The biological information contained in the genome, such as the biological processes, molecular data, and cellular components, was interpreted using Gene Ontology (GO). By connecting the function of gene products with metabolic pathways, the Kyoto Encyclopedia of Genes and Genomes (KEGG) enables the understanding of biological systems at the molecular level.

## 3. Results

### 3.1. Identification of GST Isoforms

This study aimed to identify and analyze the differential expression of each isoform or class of GST enzymes. The detection of all targeted enzymes was conducted by blasting the mass spectrometry data obtained with the *Aedes* genus database. The data obtained were further analyzed using UniProtKB up to the GST enzyme classes and each isoform was designated as its proposed identification label for easy analysis and reference. The identification of GSTs was carried out according to the International Union of Biochemistry and Molecular Biology (IUBMB). The details of the isoforms identified are summarized and presented in Table 1. In total, 24 GST isoforms were successfully identified in up to 6 different classes, which were Delta, Epsilon, Sigma, Zeta, Omega, and Iota. However, from the analysis, one unidentified gene/isoform which did not belong to any of the current GST classes was detected and designated as GSTX1-1 (24.817 kDa). For the Delta class, four isoforms of the Delta1 class (GSTD1-1 to GSTD1-4) and three isoforms of the Delta4 class (GSTD4-1 to GSTD4-3) with a molecular weight ranging from 23.146 kDa to 24.855 kDa were identified. Two isoforms of the Delta6 class (GSTD6-1 and GSTD6-2) and one isoform of the Delta11 class (GSTD11-1) were also discovered with a higher molecular weight of 28.215 kDa, 28.148 kDa, and 25.879 kDa, respectively.

For the classification of Epsilon, one of Epsilon3 (GSTE3-1), two isoforms of Epsilon4 (GSTE4-1 and GSTE4-2), one isoform of Epsilon5 (GSTE5-1), and one isoform of Epsilon6 (GSTE6-1) were discovered with a molecular weight range from 24.635 kDa to 27.264 kDa. Only one isoform of the Iota class was identified with a molecular weight of 26.177 kDa and was designated as GSTI-1. For the Omega class, only the Omega1 class was detected with four isoforms designated as GSTO1-1 to GSTO1-4 with a molecular weight ranging from 28.551 kDa to 29.618 kDa. A similar pattern was observed for the Sigma class whereby only the Sigma1 class was found with two isoforms (GSTS1-1 and GSTS1-2) and exhibited a molecular weight of 23.249 kDa and 23.216 kDa. One isoform of the Zeta1 class was identified and designated as GSTZ1-1 (26.39 kDa). Overall, a close range of 23.146 kDa to 29.618 kDa in molecular weight as well as a pI value of 5.17 to 8.51 was observed among all the classes of GST detected in the in vivo homogenized samples.

### 3.2. Protein-Protein Interaction (PPI) Network Analysis of GST Families

To predict the interacting proteins and the outcomes of the in vivo biological analysis, *Ae. albopictus* GST protein in the listed classes (Table 1) was used as the model and applied to the STRING 11.5 tool. Based on the results from 13 GSTs outputs, 10 functional partners were identified in the network analyses for GSTs, listed in Table 2 with their scores and functions. The computerized output of the 13 GSTs was reflected in the in vivo analysis earlier.

**Figure 1:**
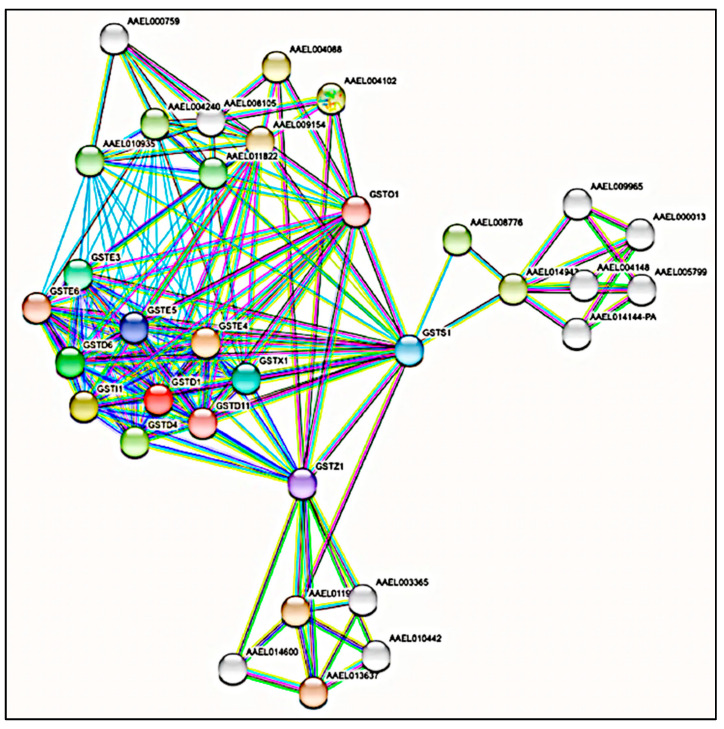
The interactive network view of *Aedes* GSTs with predicted proteins of protein-protein interactions analysis using STRING 11.5 tool at PPI enrichment *p*-value < 1.0 × 10^−16^. Network nodes are proteins, and the edges represent the predicted functional associations. The K-means algorithm was used to cluster the proteins in 13 different GST classes. Every colored node corresponds to a cluster. Inter-cluster edges are represented by dashed lines.

Furthermore, the enriched pathways of the biological process, the molecular function, and the KEGG obtained by PPI analysis for the identified GSTs are listed in Table 3 and Table 4 with their *p*-values. The K-means algorithm was used for protein clustering in 13 different colors based on the different GST classes that contributed to the analysis (Figure 1). The result of the biological processes and molecular functioning in GO showed that the protein peptides are involved in several processes. The results obtained a total of 47 terms, containing 27 biological processes and 20 molecular functions (Table 3). The glutathione metabolic process and the peptide metabolic process had the higher enrichment level among the biological processes. In terms of molecular functions, the peptides were mainly associated with Glutathione transferase activity. Catalytic activity, Carbon-halide lyase activity, Glutathione hydrolase activity, and Peptidyltransferase activity also showed a correlation between the molecular functions and the identified isoforms corresponding to the significant *p*-value (the smaller the *p*, the higher the enrichment level).

The results of the KEGG analysis of GST proteins identified 12 pathways involved in all 13 GST classes (Table 4). The enrichment results showed that the glutathione metabolism and metabolic pathways were the highest in the KEGG analysis. However, the GSTs were also associated with the drug metabolism of cytochrome P450 and other enzymes as well as the arachidonic acid metabolism. On the basis of the GO and KEGG results, our initial hypothesis was that the GSTs play an important role in xenobiotic metabolism and interacted with other enzymes in the molecule to maximize the metabolism process in the organisms.

### 3.3. Effect of Acute Insecticide Treatment on GSTs Activities

The present study used permethrin (pyrethroid) and malathion (organophosphate), which are from the common insecticide classes used in mosquito control strategies. Table 5 shows the calculated sub-lethal concentration (LC50) values obtained, which were used in acute insecticide treatments. It indicated that permethrin was more effective against *Ae. albopictus* larvae followed by malathion insecticide with LC50 values of 0.023 mg/L and 0.099 mg/L, respectively. The determined LC50s were used to investigate the effect of acute insecticide treatment on GST activities and moreover on the change in the abundance of each GST peptide. Table 6 shows the result of a 1-sample *t*-test of the total protein content (µg) of the larvae upon 24 h of acute treatment with malathion and permethrin. The total protein content of the larvae exposed to 24 h of acute permethrin and malathion treatments increased significantly (*p* < 0.05) after 24 h, at 5.3 × 10^−3^ ± 0.006 mg (1.36-fold) and 4.3 × 10^−3^ ± 0.002 mg (1.10-fold), respectively, compared to the value of the susceptible strain. The total GST activity in the larvae after 24 h of permethrin and malathion treatment increased up to 13.97 × 10^−3^ ± 0.01 μmol/min (1.84-fold) and 10.75 × 10^−3^ ± 0.02 μmol/min (1.41-fold), respectively, and were found to be significant (*p* < 0.05) upon comparison to the control strain. Similarly, the specific activity of GSTs was 2.635 ± 0.08 µmol/min/mg (1.35-fold) and 2.502 ± 0.050 µmol/min/mg (1.29-fold) for larvae under 24 h of permethrin and malathion treatments, respectively, compared to the control strain (*p* < 0.05). Conclusively, a varying increment in total GST enzyme activities and specific GST activities was detected after a 24 h period of acute malathion and permethrin treatments.

### 3.4. Differential Analysis of the Peptide Abundance under Xenobiotic Challenge

The fold-change in the peptide abundance of various isoforms of GSTs is summarized in Table 7. The elevated peptide abundance fold-change was detected from isoforms GSTD1-2, GSTD1-3, and GSTD1-4, with a fold range of 1.92-fold to 2.26-fold, followed by GSTE3-1 and GSTE4-2 under permethrin and malathion treatments. Meanwhile, the fold-change in the peptide abundance of GSTE5-1 and GSTE6-1 after 24 h of acute permethrin treatment decreased by 0.66-fold each. Only GSTS1-2 was found to decrease the peptide abundance fold-change (0.75-fold) under malathion treatment.

## 4. Discussion

### 4.1. Effect of the GST Enzymatic Activities of Aedes albopictus Larvae under 24 h of Acute Insecticide Challenges

The acute treatment of early 4th instar larvae treated for 24 h with sub-lethal concentrations of permethrin and malathion was conducted to observe the induction effect of the enzyme’s activities in the presence of toxicological challenges. Sub-lethal doses were selected for this study and defined as acute treatment because this dose has been tested to yield an optimum level of 50% of surviving larvae to be used for further analysis. A pilot study with lesser doses yielded less satisfactory enzymatic activities while higher doses resulted in a very limited number of surviving larvae and enzymatic activities which were lower than the control strain.

In insects, the metabolism of xenobiotics is one of the predominant insecticide detoxification mechanisms [35]. The biosynthesis of enzymes is suggested to occur because of a direct reaction upon exposure to xenobiotics by enzymes that hydrolyze, oxidize, and conjugate xenobiotics into less toxic, more water-soluble products thereby causing the products to be easily excreted [36,37]. The acute insecticide treatment of mosquitoes induces different levels of metabolic enzyme production which cumulatively increase the overall total protein content in the mosquito. The overall total protein fold-change of larvae post-acute malathion and permethrin treatments showed a significant increment (*p* < 0.05) upon comparison with the control strain, respectively. It has been suggested that the increase in protein content may have an impact on the increased activity of enzymes under toxicant stress, which is connected to an oxidative stress state in induced larvae. According to Devonshire et al. [38], metabolic defense mechanisms can be accelerated by the overproduction of GST content followed by an increase in its activity. Moreover, in Yan et al. [39], GSTs have been reported to be present in high concentrations in *Apis cerana* after being challenged by an extreme oxidative stress agent. The introduction of oxidative stress agents, namely insecticides such as permethrin and malathion, may lead to antioxidant defense by the initiation of the repairing activity of the damaged secondary product generated by reactive oxygen species (ROS) [33,40,41]. The determination of the total and specific enzyme activities is crucial to observe the effect of acute insecticide exposure on the rate of the metabolic enzyme activities of the test mosquitoes. In samples treated with acute malathion for 24 h, the difference in the fold between the total and specific enzyme activities of GSTs was found to be significantly elevated (*p* < 0.05) compared to the control strain. The elevation of the fold-change in the enzymatic activity of specific GSTs upon acute malathion treatment for 24 h is justifiable because it has a wide range of substrate specificity to different insecticide classes including organophosphates [42]. Upon comparison to the control strain, the elevation of the total and specific enzyme activity of GSTs (*p* < 0.05) was detected in the susceptible larvae after a 24 h acute permethrin treatment. The same pattern has been recorded in previous studies whereby there was a considerable increment in the enzymatic activities of GSTs in the field strain as well as the laboratory-selected *Ae. aegypti* mosquitoes, which implied its possible involvement in pyrethroid metabolism and resistance [43,44,45]. Apart from that, the results obtained are in line with several earlier studies on the involvement of GST enzyme activity in insecticide resistance including pyrethroid resistance [2,4,9,10]. Exposure to insecticides activates the enzymatic activities of metabolic enzymes. Upon prolonged exposure to insecticides, the enzyme production increases for the mosquito to detoxify more insecticides to survive despite the exposure to the toxicity of the insecticides. These populations of surviving mosquitoes will eventually propagate and become resistant to insecticides and hamper vector control programs.

### 4.2. Proteomic Study of Aedes albopictus Larvae upon 24 h of Acute Insecticide Treatments

Proteomics is regarded as the detailed study of proteomes which are the protein complements of the genome [46]. This approach allows for a more comprehensive study of the structure of proteins, while elucidating the diverse functions of, as well as the interactions between proteins [47]. This branch of study has also been used to analyze mosquitoes in an effort to identify and characterize the expression of the proteins of various species, tissues, and physiological stages, as well as the response to parasites, environmental conditions, and xenobiotic challenges [47,48].

Even though elevations of induced GST enzymatic activities were detected in the larvae upon 24 h of acute permethrin and malathion treatments, it was imperative to identify the presence and peptide abundance of the specific GST isoforms to determine their involvement in the detoxification of xenobiotics. Hence, a proteomic study was conducted using the crude homogenized *Ae. albopictus* larvae which had been subjected to 24 h of acute insecticide treatments. As the identification and the peptide abundance detection were completed up to the peptide and amino acid level, this proteomic data verified the GST enzymatic activity data from the previous sections.

### 4.3. Identification of GST Enzyme Classes up to the Isoform and Their Response towards Acute Insecticide Treatments

A broad range of classes and isoforms of GST enzymes were obtained after further analysis using UniProtKB. The target detoxification enzyme, GST, is the enzyme substantially responsible for the metabolism of all four major classes of insecticides [49,50,51]. The capability of GST enzymes to metabolize insecticides is of huge concern due to the fact that the elevated rate of their enzymatic activities has a tendency to result in the emergence of tolerance and eventually resistance to insecticides which threatens vector control. From the analysis, different GST isoforms were identified in this study from the control and the acute permethrin and malathion treatments, which validated their presence in the larvae of *Ae. albopictus*.

The classification of GSTs of the same classes was made if there was more than 40% identity of amino acid sequence and other properties, namely immunological properties, phylogenetic criteria, tertiary structure, chromosomal locations, and the capacity to form heterodimers [1,3,5,15]. To the best of our knowledge, only studies by Hamzah and Alias [16,33] and Hamzah et al. [52] characterized, identified, and elucidated the effects of chemical challenges on GST enzymes in *Ae. albopictus*. In the study of Hamzah et al. [52], the protein expression was determined and quantified by analyzing the peak of the gel-expressed for each visible GST spot. The TMT labeling MS-based approach was used in the present work as an essential step to increase the number of the identified proteins. As a result, out of the six classes of insect GSTs, five were successfully detected in this study and they were identified up to their specific isoforms, and one isoform from the class Iota as well as one GSTX1 were also identified.

**Figure 2:**
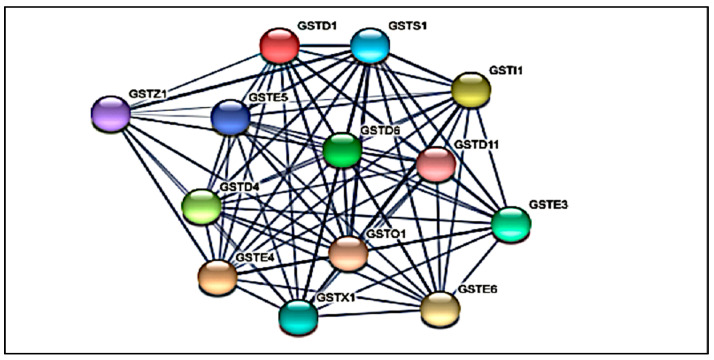
The interactive network view among *Aedes* GSTs of predicted protein-protein interaction analysis using STRING 11.5 tool. No. of nodes: 13; no of edge: 12; average node degree: 11.7; average local clustering coefficient: 0.977; PPI enrichment *p*-value < 1.0 × 10^−16^.

In this study, via protein-protein interaction analysis using STRING 11.5, all 7 GST classes were further annotated into 13 domains as shown in Figure 2. The network shows more significant intra-network interactions than expected. This means that the proteins have more interactions among themselves than what would be expected for a random set of proteins of the same size and degree distribution drawn from the genome. Such an enrichment indicates that the proteins are at least partially biologically connected as a group. In the functional enrichment network, from the Gene Ontology analysis, the 13 domains were identified under the glutathione metabolic biological process (GO:0006749, *p*-value: 1.93 × 10^−29^), and the molecular functions involved are due to glutathione transferase (GO: 0016848, *p*-value: 2.92 × 10^−8^) and carbon-halide lyase activity (GO:004364, *p*-value: 1.21 × 10^−31^). The domains were located in the subcellular cytoplasm (GO:0005737, *p*-value: 0.00015) and highly significantly involved in the glutathione metabolism (*p*-value: 5.39 × 10^−25^), metabolic pathways (*p*-value: 1.29 × 10^−14^), drug metabolism corresponding with other enzymes (*p*-value: 2.08 × 10^−19^), drug metabolism corresponding with cytochrome P450 (*p*-value: 8.55 × 10^−24^), and the metabolism of xenobiotics by cytochrome P450 (*p*-value: 8.55 × 10^−24^) with the KEGG Pathway IDs aag00480, aag01100, aag00983, aag00980, and aag00980, respectively.

The analysis further showed that the xenobiotic metabolism involved interactions not only among the domains but the interaction enrichment involved a total of 33 proteins around the input domains (Figure 1). In the analysis, STRING 11.5 then further added (by default) other occurring proteins around the network that showed expected connectivity enrichment with an average local clustering coefficient of 0.779 and a PPI enrichment *p*-value < 1.0 × 10^−16^. Generally, proteins do not work independently; this discovery lends credence to the basic proposition that proteins mediate all biological processes through interactions with other proteins, the formation of protein complexes, and the development of metabolic and signaling pathways [53,54]. Networks in biology can be established using PPI data. The special and significant nodes in the biological network may be located and the relationships between these nodes can be further understood by a thorough investigation of the network [55,56].

Furthermore, of all the isoforms identified in the samples that underwent 24 h of acute permethrin treatment, the peptide abundance of isoforms from Delta class GSTs (GSTD1-2, GSTD1-3, and GSTD1-4) were overexpressed (more than 2-fold), followed by peptide abundance of isoforms from Epsilon class GSTs (GSTE4-2). A similar but lower fold-change in the peptide abundance of isoforms was also detected in the samples that underwent 24 h of acute malathion treatment, with the highest overexpression in the peptide abundance in isoforms from the GST Delta class (GSTD1-2, GSTD1-3, and GSTD1-4 with a GO term of GO:0006749) followed by isoforms from Epsilon class which are GSTE3-1 (GO:0006749) and GSTE4-2 (GO:0016740; GO:0006749). These results showed a major involvement of Delta and Epsilon GSTs in the metabolism of insecticides. The information regarding the contribution of insect-specific Epsilon and Delta GSTs, which are the largest classes of cytosolic GSTs to insecticide detoxifications as well as resistance, has been well documented [7,13,57,58]. From their GO terms, these five isoforms have a biological and molecular function that involves the glutathione metabolic process and transferase activity. As in the chemical structure, permethrin is a synthetic insecticide that contains a cyclopropane ring which is substituted with a 2,2-dichlorovinyl and gem-dimethyl groups while malathion contains phosphate groups in the chemical structure (PubChem, NCBI). GSTE4-2 was found to be involved in the molecular function of transferase activity where the mode of action to counter the toxicological effect is by the catalytic activity of the methyl group in permethrin and the phosphate group in malathion from the donor to the acceptor to produce water-soluble metabolites that are more readily excreted (GO:0016740). Moreover, the main mode of action is by performing the glutathione metabolic biological process (GO:0006749) which acts as an antioxidant in the protection of sulfhydryl groups in enzymes and other important functional proteins, hence it could also be protective against substances such as permethrin and malathion which are known as oxidative stress inducers [52].

Moreover, overexpression in the peptide abundance of different GST isoforms suggests the involvement and induction of these isoforms upon a 24 h acute permethrin and malathion treatment and validates the outcomes attained from the biochemical assay, whereby the fold-change of the total as well as specific enzymatic activity of GSTs were also elevated in larvae that underwent 24 h of acute permethrin and malathion treatment. The GST, as the major phase II detoxification enzyme, plays a main role as a defense enzyme in the mosquito by catalyzing the conjugation process of GSH to the exposed toxic insecticides [13]. Apart from that, there have been reports about the direct involvement of GSTs in oxidative stress and in one of the mechanisms in the metabolism of insecticides [39,57,59,60]. Hence, GST overexpression would definitely increase tolerance to cytotoxicity [1].

Upon 24 h of acute permethrin treatment, the Zeta class of GSTs (GSTZ1-1) exhibited an overexpression in the fold-change of peptide abundances which implies its involvement in detoxification activities. Blackburn et al. [61] reported that a deficiency of glutathione transferase Zeta causes oxidative stress and the activation of antioxidant response pathways. There was an overall reduction in the fold-change of the peptide abundance from Sigma class (GSTS1-1 and GSTS1-2) for both the larvae that underwent 24 h of acute permethrin treatment and those that underwent 24 h of acute malathion treatment. Omega class GSTs (isoforms: GSTO1-1 to GSTO1-4) from both the larvae that underwent 24 h of acute permethrin treatment and those that underwent 24 h of acute malathion treatment showed a fold change anywhere from negligible to slightly higher compared to the control. The pattern of fold-changes suggests that these classes of GST enzymes (Sigma and Omega) did not contribute to the detoxification activities. In Figure 1, the GSTS1, GSTO1, and GSTZ1 nodes interacted significantly with proteins outside the protein group (the non-GST-related proteins). It should be noted that the involvement of the Omega and Sigma classes of GSTs in housekeeping has also been reported. A few studies indicate that apart from the Omega and Sigma classes of GST, the Zeta class also has a relatively broad taxonomic distribution and serves an essential role in housekeeping, and this information, therefore, dismisses its involvement in the metabolism of xenobiotics [62,63]. Collectively, from this study, the involvement of different classes and isoforms of GST enzymes was detected and identified in *Ae. albopictus* larvae upon 24 h of acute permethrin and malathion treatment. This study enables the pinpointing of GST enzymes that potentially underpin the development of resistance in response to pesticide challenges. Invariably, this will help in the early detection and prediction of resistance development in field strains of the vector.

## 5. Conclusions

The present study provides some biochemical and proteomic information on the targeted detoxification enzymes (GSTs) in susceptible insecticide-treated strains. Furthermore, the total protein content, total enzymatic activity, and specific enzymatic activity of GSTs were found to be significantly elevated after 24 h of acute permethrin and malathion treatment on larvae. In the proteomic study, the occurrence of multiple isoforms of GSTs in samples that underwent 24 h of acute insecticide treatment with varying fold-change in their peptide abundance was identified. Various isoforms from five classes (Delta, Epsilon, Omega, Sigma, Iota, and Zeta) as well as one unidentified GST, summing up to 24 GST isoforms, were identified. Cumulatively, the results of the peptide abundance quantitative study reflected the results of the enzymatic activities from the biochemical analysis. The in vivo peptide abundance correlated with its functional analysis (*p*-value < 1.0 × 10^−16^), suggesting that the mechanism of specific insecticide (permethrin and malathion) detoxification involved specific GST isoenzymes (Delta and Epsilon GSTs) via specific metabolic strategies (direct glutathione metabolism, the sequestration of the insecticides, or providing protection for sulfhydryl groups). The results obtained from this research provide more knowledge about the current resistance status and enzymatic activity of GST enzymes in *Ae. albopictus* mosquitoes upon acute and xenobiotic challenges. Knowledge of the resistance status and GST enzymatic activity in the vector is key to understanding the patterns of GST-mediated resistance for effective resistance management. The differentially expressed proteins could be used for monitoring and surveillance purposes to detect incipient pesticide resistance for early management in *Ae. albopictus*.

## Figures and Tables

**Table 1:** In vivo glutathione S-transferase isoforms identified in susceptible, permethrin-treated, and malathion-treated *Aedes albopictus*.

Accession	Identified Protein	Class	MW (kDa)	pI Value	Peptide No.	AA Coverage	Proposed Isoform Identification
J9HXZ8	AAEL001061-PC GN = GSTD1	Delta1	23.845	5.96	8	211	GSTD1-1
J9HHL7	AAEL001061-PB	Delta1	23.778	6.07	7	209	GSTD1-2
Q17MA9	AAEL001061-PA GN = GSTD1	Delta1	24.755	5.44	2	219	GSTD1-3
A0A1S4EXN6	Glutathione S-transferase GN = 5568355	Delta1	24.538	5.9	2	216	GSTD1-4
Q6PTY1	Glutathione S-transferase OX = 7159	Delta4	23.146	7.94	2	205	GSTD4-1
A0A0N8ES64	Glutathione S-transferase GSTD4 OX = 7159	Delta4	24.855	6.14	1	218	GSTD4-2
Q17MB8	AAEL001054-PA GN = GSTD4	Delta4	24.085	6.14	1	211	GSTD4-3
Q16SH6	AAEL010591-PA GN = GSTD6	Delta6	28.215	5.74	1	249	GSTD6-1
A0A023ENG1	Glutathione S-transferase e2 OX = 7160	Delta6	28.148	5.54	1	249	GSTD6-2
Q16SH7	AAEL010582-PA GN = GSTD11	Delta11	25.879	5.95	3	222	GSTD11-1
Q170C6	AAEL007947-PA GN = GSTE3	Epsilon3	24.824	6.54	1	222	GSTE3-1
Q5PY78	AAEL007962-PA GN = GSTe4	Epsilon4	25.03	7.11	3	224	GSTE4-1
A0A0P6IV26	Putative glutathione S-transferase e4	Epsilon4	27.264	6.81	2	244	GSTE4-2
Q170C9	AAEL007964-PA GN = GSTE5	Epsilon5	24.635	5.2	1	221	GSTE5-1
Q170C7	AAEL007946-PA GN = GSTE6	Epsilon6	24.738	5.97	2	220	GSTE6-1
Q16P53	AAEL011752-PA GN = GSTI1	Iota1	26.177	6.15	1	231	GSTI1-1
J9E9C0	AAEL017085-PA GN = GSTO1	Omega1	28.589	7.03	6	248	GSTO1-1
A0A1S4G560	Glutathione S-transferase GN = 23687505	Omega1	29.618	8.51	6	257	GSTO1-2
A0A0P6J0T5	Glutathione S-transferase OX = 7159	Omega1	29.572	8.51	6	257	GSTO1-3
Q1HQK1	Glutathione S-transferaseOX = 7159	Omega1	28.608	7.42	6	248	GSTO1-4
Q16P79	AAEL011741-PA GN = GSTS1	Sigma1	23.249	5.2	9	203	GSTS1-1
Q16P80	AAEL011741-PB GN = GSTS1	Sigma1	23.216	5.17	2	203	GSTS1-2
Q0C791	AAEL000092-PA GN = GSTX1	GSTX1	24.817	5.78	4	218	GSTX1-1
Q16NL9	AAEL011934-PA GN = GSTZ1	Zeta1	26.39	6.92	1	233	GSTZ1-1

MW: molecular weight; kDa: kilodalton, pI: isoelectric point value; AA: amino acid. All identified peptides were validated based on q-value (Percolator^®^ algorithm) with less than 5% false discovery rate.

**Table 2:** Characteristics of input proteins and functional partners in GST-class protein-protein interactions from STRING 11.5 analysis.

Input Protein	Information	
GSTD1	Glutathione s-transferase 1 isoform ×1; Glutathione S-transferase (GSTD1); Glutathione transferase (219 aa)	
GSTE4	Glutathione S-transferase e4; Glutathione transferase (224 aa)	
GSTI1	Glutathione S-transferase 1; Belongs to the GST superfamily (231 aa)	
GSTD4	Glutathione S-transferase 1; AAEL001054-PA; Glutathione transferase (211 aa)	
GSTD6	Glutathione S-transferase 1; Glutathione transferase; Belongs to the GST superfamily (249 aa)	
GSTE3	Glutathione S-transferase 1; Belongs to the GST superfamily (222 aa)	
GSTX1	Glutathione S-transferase d4; Glutathione transferase (218 aa)	
GSTS1	Prostaglandin-H2 D-isomerase/glutathione transferase; Glutathione transferase (203 aa)	
GSTE5	Glutathione S-transferase 1; Belongs to the GST superfamily (221 aa)	
GSTZ1	Probable maleylacetoacetate isomerase 2 isoform x1; Belongs to the GST superfamily (233 aa)	
GSTD11	Glutathione S-transferase 1-1; Belongs to the GST superfamily (222 aa)	
GSTE6	Glutathione S-transferase 1; Glutathione transferase (220 aa)	
GSTO1	Pyrimidodiazepine synthase; Belongs to the GST superfamily (248 aa)	
**Predicted Functional Partner**		
**Accession**	**Information**	**Score**
AAEL013637	Homogentisate 1,2-dioxygenase	0.999
AAEL011973	Fumarylacetoacetate hydrolase	0.998
AAEL009154	Glutathione synthetase isoform x1; Glutathione synthetase	0.968
AAEL004088	Aldose reductase isoform x1; Aldo-keto reductase	0.958
AAEL004102	Aldehyde reductase; Aldo-keto reductase	0.958
AAEL013637	Homogentisate 1,2-dioxygenase	0.999
AAEL011973	Fumarylacetoacetate hydrolase	0.998
AAEL009154	Glutathione synthetase isoform x1; Glutathione synthetase	0.968
AAEL004088	Aldose reductase isoform x1; Aldo-keto reductase	0.958
AAEL004102	Aldehyde reductase; Aldo-keto reductase	0.958

**Table 3:** Characteristics of biological process and molecular function of protein-protein interaction of GSTs with metabolic detoxification related protein STRING 11.5.

Biological Process (GO)			
GO-Term	Pathway Description	Count in Network	*p*-Values
GO:0000413	Protein peptidyl-prolyl isomerization	3 of 29	0.0134
GO:0006457	Protein folding	8 of 134	2.39 × 10^−6^
GO:0006518	Peptide metabolic process	21 of 471	1.25 × 10^−17^
GO:0006559	L-phenylalanine catabolic process	3 of 5	0.00028
GO:0006570	Tyrosine metabolic process	4 of 13	4.58 × 10^−5^
GO:0006572	Tyrosine catabolic process	3 of 3	0.00014
GO:0006749	Glutathione metabolic process	20 of 43	1.91 × 10^−34^
GO:0006750	Glutathione biosynthetic process	3 of 4	0.00020
GO:0006751	Glutathione catabolic process	4 of 12	3.76 × 10^−5^
GO:0006807	Nitrogen compound metabolic process	29 of 4303	2.34 × 10^−5^
GO:0008152	Metabolic process	34 of 5833	1.22 × 10^−5^
GO:0009074	Aromatic amino acid family catabolic process	5 of 12	5.50 × 10^−7^
GO:0009987	Cellular process	39 of 8701	0.00012
GO:0034605	Cellular response to heat	4 of 39	0.00016
GO:0034641	Cellular nitrogen compound metabolic process	23 of 2098	0.0011
GO:0034605	Cellular response to heat	4 of 39	4.25 × 10^−7^
GO:0043043	Peptide biosynthetic process	7 of 366	0.0093
GO:0043171	Peptide catabolic process	5 of 54	0.00017
GO:0044237	Cellular metabolic process	31 of 4455	2.26 × 10^−6^
GO:0044248	Cellular catabolic process	10 of 885	0.0164
GO:0050821	Protein stabilization	4 of 47	0.0020
GO:0071704	Organic substance metabolic process	29 of 5131	0.00063
GO:0071722	Detoxification of arsenic-containing substance	2 of 2	0.0072
GO:0098754	Detoxification	4 of 71	0.0080
GO:1901564	Organonitrogen compound metabolic process	28 of 3183	2.23 × 10^−7^
GO:1901565	Organonitrogen compound catabolic process	10 of 597	0.00081
GO:1901606	Alpha-amino acid catabolic process	5 of 60	0.00024
**Molecular Function (GO)**			
GO:0000048	Peptidyltransferase activity	4 of 10	1.57 × 10^−5^
GO:0003755	Peptidyl-prolyl cis-trans isomerase activity	3 of 30	0.0089
GO:0003824	Catalytic activity	33 of 4721	2.22 × 10^−7^
GO:0003868	4-hydroxyphenylpyruvate dioxygenase activity	2 of 2	0.0049
GO:0004032	alditol:NADP+ 1-oxidoreductase activity	2 of 11	0.0373
GO:0004364	Glutathione transferase activity	13 of 30	4.08 × 10^−21^
GO:0008144	Drug binding	3 of 16	0.0027
GO:0016018	Cyclosporin a binding	2 of 9	0.0283
GO:0016702	Oxidoreductase activity, acting on single donors with incorporation of molecular oxygen, incorporation of two atoms of oxygen	3 of 16	0.0027
GO:0016740	Transferase activity	17 of 1385	1.57 × 10^−5^
GO:0016823	Hydrolase activity, acting on acid carbon-carbon bonds, in ketonic substances	2 of 2	0.0049
GO:0016848	Carbon-halide lyase activity		4.96 × 10^−6^
GO:0016853	Isomerase activity	6 of 108	0.00012
GO:0016859	Cis-trans isomerase activity	4 of 32	0.00044
GO:0031072	Heat shock protein binding	3 of 42	0.0199
GO:0033218	Amide binding	5 of 138	0.0049
GO:0036374	Glutathione hydrolase activity	4 of 10	1.57 × 10^−5^
GO:0042277	Peptide binding	4 of 105	0.0180
GO:0050220	Prostaglandin-E synthase activity	2 of 3	0.0069
GO:0051879	Hsp90 protein binding	2 of 10	0.0327

Count In Network: The first number indicates how many proteins in the network are annotated with a particular term. The second number indicates how many proteins in total (in the network and in the background) have this term assigned; *p*-value: the false discovery rate which corrected for multiple testing within each category using the Benjamini–Hochberg procedure to measure how significant the enrichment is.

**Table 4:** Characteristics of KEGG pathways of protein-protein interaction of GSTs with STRING 11.5.

KEGG Pathway			
Pathway ID	Pathway Description	Count in Network	*p*-Values
aag00051	Fructose and mannose metabolism	2 of 23	0.0260
aag00052	Galactose metabolism	2 of 28	0.0343
aag00270	Cysteine and methionine metabolism	3 of 27	0.0011
aag00350	Tyrosine metabolism	5 of 27	3.81 × 10^−7^
aag00430	Taurine and hypotaurine metabolism	4 of 7	3.02 × 10^−7^
aag00480	Glutathione metabolism	21 of 74	2.66 × 10^−34^
aag00590	Arachidonic acid metabolism	8 of 17	4.27 × 10^−14^
aag00790	Folate biosynthesis	3 of 33	0.0017
aag00980	Metabolism of xenobiotics by cytochrome P450	11 of 53	3.76 × 10^−16^
aag00982	Drug metabolism—cytochrome P450	11 of 52	3.76 × 10^−16^
aag00983	Drug metabolism—other enzymes	10 of 80	7.93 × 10^−13^
aag01100	Metabolic pathways	30 of 1039	5.51 × 10^−24^

Count In Network: The first number indicates how many proteins in the network are annotated with a particular term. The second number indicates how many proteins in total (in the network and in the background) have this term assigned; *p*-value: the false discovery rate which corrected for multiple testing within each category using the Benjamini–Hochberg procedure to measure how significant the enrichment is.

**Table 5:** *Aedes albopictus* mortality under malathion and permethrin treatments along with the calculated sub-lethal dosage (LC50).

Insecticide	Concentration (mg/L)	Mortality (%)	LC50 (mg/L)	95% Confidence Limit (mg/L)		Slope ± SD
				Lower Bound	Upper Bound	
Malathion	0.024	0				
	0.053	15				
	0.072	30				
	0.115	60	0.099	0.090	0.108	3.692 ± 0.228
	0.25	94				
	0.40	100				
Permethrin	0.002	0				
	0.006	10				
	0.018	40	0.023	0.019	0.027	3.482 + 0.223
	0.034	65				
	0.120	95				
	0.203	100				

The LC50 values were calculated statistically from the data collected from the WHO bioassays using the Probit analysis software SPSS version 24.

**Table 6:** Mean of total protein contents (mean ± SD), total GST enzyme activities (mean ± SD), and specific GST enzyme activities (mean ± SD) in *Aedes albopictus* larvae upon 24 h of acute treatment by permethrin and malathion sub-lethal dosages (LC50).

Treatment	Treatment Duration	Total Protein Contents (mg)	PF	Total Activity (μmol/min)	TAF	Specific Activity (μmol/min/mg)	SAF
Permethrin	Control 24 h	3.9 × 10^−3^ ± 0.001 5.3 × 10^−3^ ± 0.006 *	1.00 1.36	7.59 × 10^−3^ ± 0.01 13.97 × 10^−3^ ± 0.01 *	1.00 1.84	1.947 ± 0.08 2.635 ± 0.08 *	1.00 1.35
Malathion	Control 24 h	3.9 × 10^−3^ ± 0.001 4.3 × 10^−3^ ± 0.002 *	1.00 1.10	7.59 × 10^−3^ ± 0.04 10.75 × 10^−3^ ± 0.02*	1.00 1.41	1.947 ± 0.08 2.502 ± 0.05 *	1.00 1.29

Mean of total protein content, total activity, and specific activity with an asterisk (*) shows a significant difference at *p* = 0.05. The *t*-test was performed to compare the total protein contents as well as the enzyme activities between each acute insecticide-treated strain with susceptible strain only. SD: standard deviation, calculations were made per individual. PF: total protein content fold-change. TAF: total GST enzymatic activity fold-change. SAF: specific GST enzymatic activity fold-change. Fold-change calculations were calculated by dividing the values of each strain by its control counterpart. *n* = 100.

**Table 7:** Peptide abundance response of glutathione s-transferase isoforms in *Aedes albopictus* larvae upon 24 h of acute permethrin and malathion treatments.

		Peptide Abundance			Fold-Change	
No	Proposed Identification	Control	Permethrin	Malathion	Permethrin	Malathion
1	GSTD1-1	155.8	180.2	155.3	1.16	1.00
2	GSTD1-2	48.5	107.3	102.4	2.21	2.11
3	GSTD1-3	27.6	62.5	53.1	2.26	1.92
4	GSTD1-4	27.6	62.5	53.1	2.26	1.92
5	GSTD4-1	162.8	176.5	188.8	1.08	1.16
6	GSTD4-2	78.5	95.8	90.1	1.22	1.15
7	GSTD4-3	78.5	95.8	90.1	1.22	1.15
8	GSTD6-1	173.3	174	252.6	1.00	1.46
9	GSTD6-2	173.3	174	252.6	1.00	1.46
10	GSTD11-1	147.3	126	131.4	0.86	0.89
11	GSTE3-1	148	192	227.2	1.30	1.54
12	GSTE4-1	198.3	217.7	183.9	1.10	0.93
13	GSTE4-2	143.1	230.1	226.7	1.61	1.58
14	GSTE5-1	175.6	116.1	191.8	0.66	1.09
15	GSTE6-1	175.6	116.1	191.8	0.66	1.09
16	GSTI1-1	198.9	224.4	176.7	1.13	0.89
17	GSTO1-1	47.6	47	53.8	1.00	1.13
18	GSTO1-2	47.6	47	53.8	1.00	1.13
19	GSTO1-3	47.6	47	53.8	1.00	1.13
20	GSTO1-4	47.6	47	53.8	1.00	1.13
21	GSTS1-1	165.5	149.4	139	0.90	0.84
22	GSTS1-2	166.4	136.9	124.6	0.82	0.75
23	GSTX1-1	87.8	109.4	110.9	1.25	1.26
24	GSTZ1-1	16.4	20.1	19.4	1.23	1.18

GST: Glutathione S-transferase, D: Delta, E: Epsilon, I: Iota, O: Omega, S: Sigma, Z: Zeta. Fold-change of peptide abundance in acute permethrin and malathion treatment was obtained by dividing its peptide abundance value by the peptide abundance value of the control (susceptible).

## Data Availability

Data is contained within the article except spectra data which is available on request from the corresponding author.
